# Editors’ Table

**Published:** 1857-07

**Authors:** 


### Editors' Table.

MINERAL SPRINGS.—Explain it as we may, there is great medicinal value in the use of the waters of mineral springs, both internally and externally, as a drink and for bathing. We will go farther, and assert, that in proportion as we enlarge the boundaries of therapeutics, and establish clear indications for the employment of different remedies, so shall we more readily and frequently have recourse to these waters. It is not our intention to treat the subject either methodically or in detail, in this department of our journal, where we desire to talk to our readers on a variety of topics not easily classed under the usual head of medical science and practice, rather than dissert on any subject in the commonly recognized list.

The natural history of mineral springs is itself a curious and at the same time an instructive study, bringing us, as it does, at once in relation with geology, mineralogy, chemistry, and in degree, with botany. Thus, the successive changes constituting the mineralization of water from the time that this fluid falls from the atmosphere in the shape of rain, or is condensed and precipitated as dew, on the surface of the earth, and first percolates the upper strata, and then penetrates deeper through fissures in rocks, and accumulates often in subterranean lakes, or flows as subterranean rivers, until it rises again as a spring, impregnated with the earthy and saline matters of the rocks through which it had permeated, lead us through inquiries bearing on all the above branches of natural science. The thermalization of water, the depth at which it takes place, and the permanency of its temperature, when flowing out as a thermal spring, are also questions of moment, the solution of which is mixed up with a study of the existence of the central heat of our globe, exceeding that of common incandescence, and of the vents which are found for these central fires by volcanoes, and the irregular throes of earthquakes. How water penetrating to great depths becomes vaporized, how this vapor, in some cases, in its rise becomes condensed in water, but still water of a high temperature, or how in others, it serves to heat the rocks through which the veins of water pass, so that in both instances the fluid which reaches the surface flows out as a thermal spring, are also parts of the problem. Pushing our inquiries farther in the same channel, we shall be tempted to ask, whether, by a process analogous to the distillation of water into vapor, there may not be carried on, simultaneously with the latter, and by the same agency of high heat, a sublimation of various earthy and mineral substances and their volatilization, and mixture, for the time being, with the vapor of water so as to constitute a mineral watery vapor, which when condensed would become a true mineral water. We can readily conceive how, at other times, the sublimation of mineral substances should take place separately, and that in their ascent, they should be condensed as they come into contact with the cold rocks above, and leave deposits in their fissures and canals through which water subsequently percolating becomes mineralized, and furnishes a supply for mineral springs. Nearly all the constituents of the latter have been found in the form of sublimates, such as the chloride of sodium, muriate of lime, sulphate of lime, sulphur, iron, &c.

If we inquire into the composition of the water of springs, we shall find that all have, to a greater or less extent, a mineral or saline impregnation. Both river and spring water contain quite a number of substances, both solid and gaseous. The first, among the earths, are lime, magnesia, strontium, and lithia; among the alkalies, potash, soda, and ammonia; the acids, nitric and sulphuric; the metals, iron; other separate principles, iodine, bromine, and chlorine. The gaseous additions are oxygen, nitrogen, and carbonic acid, and not unfrequently sulphuretted hydrogen. It must not be supposed, however, that all or even any considerable number of these substances, are ever found in the same spring, or, if found, the substance is, in most instances, in a barely appreciable quantity. We do not speak of the combination of these several substances, as of acids with earths, oxygen with metals, &c., in the mode of combination in which they exist in the water of the spring. In mineral springs proper, we find in addition to the above list the following substances, viz., hydrogen and carburetted hydrogen gases, boracic, crenic, fluoric, formic, hydrochloric, and phosphoric acids, manganese, zinc, arsenic, antimony, copper, tin, cobalt, titanium, and zirconium, and an organized substance, called, at first, glairine, now more generally known as baregine.

It is a curious and highly important fact, that mineral waters contain not only all the chief, but also, a large number of the secondary substances which, in their simple or compound state, make up not only the surface, but the crust of the earth. Another curious coincidence deserves to be mentioned on the present occasion. It is, that the sublimates, or substances of volcanoes converted into a gaseous and fluid state, give us nearly all the ingredients of mineral and thermal springs. In the economy of nature these latter, and especially the thermal division, perform an important part, analogous to that of volcanoes, by affording multiplied vents for the escape of various gases, and especially the carbonic acid, from the deep-seated portion of the earth's crust, and thus prevent the commotions and disruptions which still, but much more rarely than without these springs, occur in the form of earthquakes. Many thermal, and at the same time mineral springs appear to be only the condensation of vapors and gases effected in portions of fissures from volcanic vents, when the temperature becomes sufficiently lowered. In another point of view, thermal springs cannot but exert a considerable effect on the temperature of the upper strata of the earth's crust and on vegetation, by the vast reticulation which they form, and the considerable and constant evolution of heat, in their subterranean course.

We had intended to notice some of what may be called the Curiosities of Mineral Springs, such as the conversion of the snow and ice into thermal springs in volcanic districts (Iceland for example), the emergence of hot springs from beneath banks of ice, sometimes, also from the beds of rivers; the close proximity of hot and cold springs; the immense amount of saline substances in solution, given out by some mineral springs, and the extensive calcareous deposits from others; the eruption and accompanying noises by the issue of the waters of some in Iceland, St. Michael, &c.; but we must defer these until our next talk on the subject. Two instances we may, however, mention of the quantity of saline substances given out with the water of mineral springs. The first is at Carlsbad, the issue from the hot springs of which amounts, annually, to 746,884 pounds of carbonate of soda, and to 1,132,923 pounds of sulphate of soda, without reckoning the other substances which accompany these salts. The second is at Vichy, the hot springs of which give out a million of pounds of the carbonate of soda.*

---

PENNSYLVANIA STATE MEDICAL SOCIETY.—In another part of the Journal will be found an account of the proceedings of this Association, whose

* We would gladly have said something in relation to the employment, therapeutically, of the mineral springs of our own country, but are precluded from doing so by want of room. We may remind our readers, however, of the volume by Dr. Bell, on "The Mineral and Thermal Springs of the United States and Canada;" and that by Dr. Moorman on "The Virginia Springs," which with its map and plates, and the routes and distances to the various springs, and an account of the Natural Curiosities of Virginia, will be found an instructive companion to invalids and travellers in that region. Dr. Burke's book on the "Mineral Springs of Virginia," may also be consulted with advantage.

meeting we had the pleasure to attend, although not until the second day of its session. Upon our arrival, which was late in the morning, we found Dr. Worthington, of Westchester, one of the late Vice-Presidents, in the chair, his countenance beaming with intelligence and benevolence. The address of the President, Dr. La Roche, had been delivered on the previous day, and was spoken of as a chaste, beautiful, and forcible production, every way worthy of the author of the great work on yellow fever. A short time before the adjournment of the meeting, Dr. John L. Atlee, the President elect, was conducted to the chair, and, in a few feeling and pertinent remarks, took his seat. The Society may consider itself as peculiarly fortunate in having selected so able and zealous a presiding officer. Without wishing to make any invidious distinctions, we may venture to affirm that there is not a member of the Association who has evinced a deeper interest in its welfare, or taken a more active part in its deliberations. Besides, Dr. Atlee was eminently entitled to the honor by his age and by his position, as one of the most distinguished physicians of the State. From what we could learn, the meeting was characterized throughout by the most perfect harmony and good feeling.

In the afternoon, after the adjournment of the meeting, the members were escorted to the neighborhood of Westchester, to view the battle-ground of the Brandywine, celebrated in history, and rendered classical as one of the most bloody scenes of the Revolution. As we rode along, over a country almost indescribably beautiful, the various places of special interest, as Osborn's Heights, Birmingham Meeting-House, the Headquarters of Genls. Washington, Howe, and Lafayette, Cheney's Fort, and the "dark and bloody field," were pointed out to us by our venerable guide, Dr. Darlington, now the oldest, as he has long been the most distinguished, citizen of Westchester. It was pleasant to listen to the "old man eloquent," as he rehearsed, with animated face and sparkling eye, some of the stirring incidents which befell our poor countrymen on that melancholy day, when the "red coats" killed three hundred of our best troops and wounded six hundred more. The afternoon's sun had sunk far below the horizon when we reached our lodgings, to prepare for the evening's entertainment, given by the Medical Society of Chester County, at "Cabinet Hall." The table was spread in beautiful taste, and the rapidity with which the various articles under which it groaned disappeared, afforded ample evidence of their appreciation. After two hours spent in agreeable and delightful intercourse with each other, and the invited guests, embracing a number of the most distinguished citizens of the place, the members retreated to their respective apartments, feeling better, if not wiser, for having been assembled together, and deeply regretting that so charming a reunion should have been so short.

Westchester is a beautiful town, situated in one of the most fertile and lovely regions of the State, and distinguished for its excellent schools, its Academy of Natural Sciences, its able and united medical faculty; and, above all, as the residence of one of the greatest botanists of the age. Who has not heard of William Darlington, the great *savant* of this charming spot, rendered famous by his botanical researches and his long citizenship? His "Flora Cestrica" has made his name familiar to all the scientific men of America and Europe. He has an immense herbarium, the result of many years' labor, and of a vast correspondence with the botanists of nearly all parts of the world. He began the study of botany early in life, and has remained faithful to it up to the present moment, having had his tastes strengthened and confirmed by Professor Benjamin Smith Barton, of the University of Pennsylvania, of which he is a graduate. Soon after he entered the profession, he served as a major in the "late war" between this country and Great Britain, and, upon quitting the army, was elected a member of Congress, in which he remained six years. After retiring to private life, he devoted himself to the practice of medicine and the exploration of the botany of Chester County, at length embodying the results of his observations in the admirable work above alluded to, and which reached its third edition in 1853.

Of the floral resources of Chester County an idea may be formed when it is stated that they are fully two-thirds as great as those of all Great Britain. Of this fact we had no idea, until we were informed of it by Dr. Darlington. This may, perhaps, in some degree, account for the wonderful success which has attended his labors in this beautiful field of nature. Another circumstance, however, which has powerfully contributed to this result, has been his own innate love of the science, and his determination to make it the great and absorbing study of his life. We cannot forbear, in concluding this brief notice of our delightful visit to West Chester, and the few pleasant hours we spent in the company of this worthy man, to quote from the preface to the last edition of his Flora, the following beautiful passage, and to express the hope that the evening of his life may be as tranquil and joyous as the morning and noon were pure, active, and useful. We can almost imagine that when he shall be called hence, the flowers of Chester will unite in a body to send him their choicest wreath to deck his brow, and a pillow of roses and honeysuckle to support his head.

"He has devoted," says the author, "the leisure hours of a number of years to the favorite employment of endeavoring to excite a taste for the study of plants, and to aid the researches of his juvenile contemporaries in that charming department of Natural Science. His efforts have been amply repaid by the gratification attending the communion of kindred spirits, and such is his delight in the pursuit that, sometimes, in his dreamy reveries, he indulges the flattering idea, that if, peradventure, his work should survive him, he may continue to be an humble auxiliary of our youthful botanists; and, in some sort, a companion of their studies, even when the flowers of *Chester* shall be blooming on his grave."

---

AMERICAN MEDICAL ASSOCIATION.—It will be seen by reference to the proceedings of this body that the late meeting was but poorly attended, and in the matter of reports, which, at last, is the only means by which the success of the organization can or should be judged, proved almost a failure. We urge no objection to the character of the reports, indeed we have reason to believe that some of them, and more especially the two prize essays, possess unusual merit; but what we take exception to is the fewness of their number. Of nineteen special committees appointed at the last meeting only three were ready to report; and of the twenty or thirty standing committees on Medical Topography and Epidemics, only one. In a pecuniary point of view, it may be as well that the next volume of Transactions will be small, for as the treasury depends for its supply mainly upon the assessment of the delegates, an attendance of one hundred and eighty, which, we understand, was the total number at Nashville, would hardly furnish means sufficient to print a volume one-third the size of the bulky affair that was issued last year.

There seems to be but one opinion in regard to the reception which the members met with from the profession and citizens, generally, of Nashville. In point of open, warm-hearted hospitality, and genuine refinement, the dwellers in the "City of Rocks" are unsurpassed by any community in the land; and we learn from various sources that they left nothing unthought of to minister to the comfort and luxury of the delegates.

---

Quarantine Convention.—We are glad to learn that this Convention, the first of its kind in America and the second anywhere, has succeeded beyond the expectations of its friends, and may be regarded as the established commencement of a series of authoritative deliberations, which must lead to inestimable public benefit.

The increasing necessity for a more general diffusion of clear and practical ideas upon questions of public hygiene, and for the consequently better administration of health-protecting measures, is too manifest, in these days of sanitary blue-books, to need urging on the professional inquirer. The example of our transatlantic neighbors, especially as shown in the growing and richly productive efforts of our British brethren, has become too strong to be resisted by the most careless medical reader. Even among our unprofessional citizens, the waves of European agitation of the subject have reached the well-informed; while the thoughtful and benevolent of all classes of our countrymen have had their fears and sympathies aroused, by the appalling experience of recent epidemics, into an activity which needs and awaits only the requisite scientific guidance to direct it into an available channel.

Some degree of success therefore was reasonably to be looked for, and has been richly won. The manner in which the invitation for the meeting was responded to, not only does great honor to the various bodies represented, and to participants in the Convention, but secures the future interest and co-operation of many doubting or indifferent parties, whose experience and opportunities should have led them to take an earlier and more encouraging position in the van of what promises to be an unequivocal reform.

Great praise is due to Dr. Jewell, the projector of the movement; to the Philadelphia Board of Health, which so handsomely and efficiently supported him; and to the Philadelphia delegation, as a Committee of Arrangements,—for the manner in which the body was received, and enabled to organize and conduct its action so readily to something like a positive demonstration.

The superior character of the medical delegates, and the evidently high position of the unprofessional representatives, secured to the Convention a weight and respectability which the importance of the occasion and object fully demanded, but which many sinister predictions had not allowed us to anticipate.

The proceedings were characterized by a harmony and business-like steadiness of purpose and celerity of action, as well as practical learning and intelligence, which could not fail to produce some valuable and decided results.

In this respect they have effected more than we had reason to expect at the outset of such an enterprise. The report of the Business Committee affords us something considerably better than a mere inauguration of a Quarantine and Sanitary Association,—something materially beyond the mere announcement of work hereafter to be done, which was the utmost that was confidently hoped for from the primary meeting. It presents us with a great deal of important precept, as admitted after careful examination and now authoritatively expressed, for the instruction of the whole community.

This action of the Convention, the nature of the propositions mooted, the almost entire unanimity with which they were adopted, and the commanding influence of the delegates, and of the bodies represented, have already made a marked impression on the country. We doubt not that this impression will grow deeper and deeper as the official record is more generally read, and as the time for the next annual assemblage approaches. We are no less certain that the second meeting will prove a large and imposing one, although not more able or select in its composition than its predecessor; and we shall look with satisfaction for the continuance of labors well begun, which cannot fail to exercise an immediate as well as lasting influence on the interests which most nearly concern the business and bosoms of every member of the Commonwealth.

---

The recent announcement of the discovery of the excito-secretory subsystem of the spinal nerves by Dr. Marshall Hall, of London, has called forth a letter, and various accompanying documents, addressed to that gentleman, from Dr. Henry F. Campbell, of Augusta, Georgia, who clearly sets forth his own claims to the discovery in question. The essential portions of Dr. Campbell's communication are copied into the London Lancet (May 2,) by the request of Dr. Marshall Hall, who gracefully yields the credit of the *idea* and the *designation* of the excito-secretory action to our countryman.

---

COMPLIMENTARY.—The article by Dr. S. M. Bemiss, of Louisville, on "Marriages of Consanguinity," published in the first number of this journal, has been copied entire by "The Journal of Psychological Medicine and Mental Pathology," edited by Dr. Forbes Winslow, of London, a periodical which has long held the very first rank in the world among publications devoted to psychological medicine. "The Dublin Medical Free Press," also makes large extracts from the same, coupled with a complimentary notice of the valuable contributions which American physicians are making to the medical sciences.

Dr. Bemiss may now hope to have his paper noticed by some one, or more of the medical periodicals of our own country.

---

WHOLESALE PLAGIARISM.—It will be seen by reference to a bibliographical notice contained in this number of the Review, that an Englishman by the name of Hogg, has very quietly appropriated a large part of Dr. Wythe's deservedly popular book, entitled "The Microscopist," to the construction of a book upon a similar subject, in Great Britain.

---

We learn that at the recent meeting of the Army Medical Board, at New York, about twenty-five applicants presented themselves for examination, of which number five were reported to the War Department, as qualified for appointment in the Medical Staff. The following list comprises the names of the successful candidates, and of the medical schools from which they received their diplomas.

Dr. Robert Bartholomew, of Maryland, graduate of the University of Maryland; Dr. J. C. Bailey, of Pennsylvania, graduate of the Medical Department of Pennsylvania College; Dr. J. C. McKee, of New York, graduate of the University of Pennsylvania; Dr. K. Ryland, of Missouri, graduate of the Missouri Medical College; Dr. W. A. Carswell, of South Carolina, graduate of the Charleston Medical College.

---

We are pleased to be able to state that the Philadelphia College of Medicine, which has heretofore always held two commencements in the year, has determined hereafter, to conform to the general custom of graduating students only at the end of the winter session.

---

We regret to learn that Dr. Huston, Professor of Materia Medica and Therapeutics in the Jefferson Medical College, has been compelled, on account of ill-health, to resign his chair. Dr. Huston was connected with the school for eighteen years, and contributed much by his talents, learning, industry, and amiable deportment, in elevating it to the unexampled prosperity which it has so long enjoyed. In 1839 and 1840, he lectured, as we are informed, with distinguished ability, on Obstetrics, having succeeded the late Dr. Samuel McClellan. On the reorganization of the College, in 1841, he became the occupant of the chair of Materia Medica and Therapeutics, the duties of which had been ably discharged, during the past two years, by Professor Dunglison, in connection with those of the chair of Physiology, at a time when there were only six professors.

Dr. Huston carries with him, into his retirement, the best wishes of his colleagues for the speedy restoration of his health and for his future prosperity and happiness.

---

Prof. G. M. Newton has resigned the chair of Anatomy in the Medical College of Georgia, at Savannah, and Prof. H. F. Campbell has been transferred to the same. The professorship of Surgical, Comparative, and Microscopic Anatomy, lately held by Dr. Campbell, has been abolished.

---

Dr. S. G. Armor has resigned the professorship of Pathology and Clinical Medicine, in the Medical College of Ohio.

---

Drs. Purple and Bulkley have retired from the editorship of the New York Journal of Medicine, leaving Dr. Stephen Smith to carry on the work alone.

---

With all due respect to the editors of the New Orleans Hospital Gazette, the only reply that we deem proper to make to the questions propounded to us in the April number of that Journal, is the simple statement contained in the publishers' circular upon the back of the Review, that "no sectional clique or *school* influence" will be represented in its pages. We wish to have it distinctly understood that our journal is not a medical school bulletin, as the article commented upon by the New Orleans Gazette sufficiently proves.

---

Boston Notions upon the editing of British Medical Books by American Physicians.—We find in the Boston Medical and Surgical Journal (May 14), over the well-known initials of the facetious Oliver Wendell Holmes, the following sprightly paragraphs, preliminary to a notice of Todd and Bowman's "Physiological Anatomy and Physiology of Man." We take especial pleasure in quoting them, not simply because of the humor they contain, but as confirmatory testimony against the practice they so handsomely condemn.

"It is always a pleasure to receive, to read, to notice, to commend a genuine book. And such a book is this before us, which, after twelve years' waiting, has at length crowned its tardy pages with the Finis and the Index.

"It may be asked what we mean by a genuine book. Let us say, in the first place, what we do not mean. We do not mean one which is written by a teacher, because he knows he can turn an honest penny by making his class buy it. Nor one which is put together of old materials for the sake of the advertisement afforded by its title-page. Still less one which, having been so patched up by some foreign drudge, is caught at by an 'American Editor,' who having added thereto a note of interrogation in brackets, a 'Qu.? Am. Ed.,' a questionable item from a German Journal (quoted from the Periscope of a well-known Reprint)—a Preface, large-typed and double-leaded, and finally the Editorial Name, is paraded before us in that pale or peppered sheepskin which is the chosen livery of these parasites,—the acari bred by the itching palms of publishers, the guinea-worms that have so burrowed into our Medical Literature.

"A genuine book is one written by a man (*ou autre*) because he knows something worth telling that has not been told. Of twenty books or of twenty professorial lectures, possibly one may meet this definition. In all the others, the writer or speaker does not write or speak because he knows, but knows solely in order that he may write or speak. The *vis a tergo* is to the *vis a fronte* in the ratio of one to twenty. Teachers who are also authors are particularly given to be thus dragged into book-making by their position, instead of being pushed into it by the stores they have heaped up and must get rid of. It is a comfort to lift a book as heavy as this one, and to feel that every page is full of honest investigation and sound thought. It is a delight to have such a noble work without any parasitical addenda clinging to it—no pediculus in the title-page—no pulex in the shape of a teasing foot-note—no ascaris in the form of an appendix. Twelve years of labor spent by two true men, observers and thinkers, did not require twelve hours revision to fit their product for the eyes of American readers. Physiology and anatomy do not grow so much older in a fortnight, that the sheets printed in London are obsolete when they arrive at New York. A few degrees of longitude and latitude do not change the laws of life, as they do (or ought to) the laws of light in Mr. Hedgecock's quadrant."

